# Genome-Wide Association Study Identifies Two Novel Regions at 11p15.5-p13 and 1p31 with Major Impact on Acute-Phase Serum Amyloid A

**DOI:** 10.1371/journal.pgen.1001213

**Published:** 2010-11-18

**Authors:** Carola Marzi, Eva Albrecht, Pirro G. Hysi, Vasiliki Lagou, Melanie Waldenberger, Anke Tönjes, Inga Prokopenko, Katharina Heim, Hannah Blackburn, Janina S. Ried, Marcus E. Kleber, Massimo Mangino, Barbara Thorand, Annette Peters, Christopher J. Hammond, Harald Grallert, Bernhard O. Boehm, Peter Kovacs, Ludwig Geistlinger, Holger Prokisch, Bernhard R. Winkelmann, Tim D. Spector, H.-Erich Wichmann, Michael Stumvoll, Nicole Soranzo, Winfried März, Wolfgang Koenig, Thomas Illig, Christian Gieger

**Affiliations:** 1 Institute of Epidemiology, Helmholtz Zentrum München, German Research Center for Environmental Health, Neuherberg, Germany; 2 Department of Twin Research and Genetic Epidemiology, King's College London, London, United Kingdom; 3 Oxford Centre for Diabetes, Endocrinology, and Metabolism, University of Oxford, Oxford, United Kingdom; 4 Wellcome Trust Centre for Human Genetics, University of Oxford, Oxford, United Kingdom; 5 Department of Medicine, University of Leipzig, Leipzig, Germany; 6 Coordination Centre for Clinical Trials, University of Leipzig, Leipzig, Germany; 7 Institute of Human Genetics, Helmholtz Zentrum München, German Research Center for Environmental Health, Neuherberg, Germany; 8 Wellcome Trust Sanger Institute Genome Campus, Hinxton, United Kingdom; 9 LURIC, Freiburg, Germany; 10 Division of Endocrinology, Department of Medicine, University of Ulm, Ulm, Germany; 11 Interdisciplinary Centre for Clinical Research, Department of Internal Medicine III, University of Leipzig, Leipzig, Germany; 12 Institute of Human Genetics, Klinikum Rechts der Isar, Technische Universität München, Munich, Germany; 13 Cardiology Group Sachsenhausen, Frankfurt, Germany; 14 Institute of Medical Informatics, Biometry and Epidemiology, Chair of Epidemiology, Ludwig-Maximilians-Universität, Munich, Germany; 15 Klinikum Grosshadern, Munich, Germany; 16 SynLab Medizinisches Versorgungszentrum Heidelberg, Eppelheim, Germany; 17 Institut für Public Health, Sozialmedizin und Epidemiologie, Medizinische Fakultät Mannheim der Universität Heidelberg, Mannheim, Germany; 18 Clinical Institute of Medical and Chemical Laboratory Diagnostics, Graz, Austria; 19 Department of Internal Medicine II – Cardiology, University of Ulm Medical Center, Ulm, Germany; Georgia Institute of Technology, United States of America

## Abstract

Elevated levels of acute-phase serum amyloid A (A-SAA) cause amyloidosis and are a risk factor for atherosclerosis and its clinical complications, type 2 diabetes, as well as various malignancies. To investigate the genetic basis of A-SAA levels, we conducted the first genome-wide association study on baseline A-SAA concentrations in three population-based studies (KORA, TwinsUK, Sorbs) and one prospective case cohort study (LURIC), including a total of 4,212 participants of European descent, and identified two novel genetic susceptibility regions at 11p15.5-p13 and 1p31. The region at 11p15.5-p13 (rs4150642; p = 3.20×10^−111^) contains serum amyloid A1 (*SAA1*) and the adjacent general transcription factor 2 H1 (*GTF2H1*), Hermansky-Pudlak Syndrome 5 (*HPS5*), lactate dehydrogenase A *(LDHA)*, and lactate dehydrogenase C (*LDHC*). This region explains 10.84% of the total variation of A-SAA levels in our data, which makes up 18.37% of the total estimated heritability. The second region encloses the leptin receptor (*LEPR*) gene at 1p31 (rs12753193; p = 1.22×10^−11^) and has been found to be associated with CRP and fibrinogen in previous studies. Our findings demonstrate a key role of the 11p15.5-p13 region in the regulation of baseline A-SAA levels and provide confirmative evidence of the importance of the 1p31 region for inflammatory processes and the close interplay between A-SAA, leptin, and other acute-phase proteins.

## Author Summary

An elevated level of acute-phase serum amyloid A (A-SAA), a sensitive marker of the acute inflammatory state with high heritability estimates, causes amyloidosis and is a risk factor for atherosclerosis and its clinical complications, type 2 diabetes, as well as various malignancies. This study describes the first genome-wide association study on baseline A-SAA concentrations. In a meta-analysis of four genome-wide scans totalling 4,212 participants of European descent, we identified two novel genetic susceptibility regions on chromosomes 11 and 1 to be associated with baseline A-SAA concentrations. The chromosome 11 region contains the serum amyloid A1 gene and the adjacent genes and explains a high percentage of the total estimated heritability. The chromosome 1 region is a known genetic susceptibility region for inflammation. Taken together, we identified one region, which seems to be of key importance in the regulation of A-SAA levels and represents a novel potential target for the investigation of related clinical entities. In addition, our findings indicate a close interplay between A-SAA and other inflammatory proteins, as well as a larger role of a known genetic susceptibility region for inflammatory processes as it has been assumed in the past.

## Introduction

Serum amyloid A (SAA) is a sensitive marker of the acute inflammatory state. Its isoforms are expressed constitutively (C-SAA) and show a rapid (up to 1000-fold) increased expression in response to inflammatory stimuli such as trauma, infection, injury, and stress during the acute phase (A-SAA) [1]. The high inductive capacity along with a high conservation of genes and proteins throughout evolution of vertebrates and invertebrates suggests that A-SAA plays a key role in pathogen defence and probably functions as an immune-effector molecule [1]. Acute inflammation has mainly beneficial effects in restoring homeostasis. However, in recent years, clinical and epidemiological studies have gathered substantial evidence that A-SAA is associated with obesity [2] and that prolonged and recurrent chronic infection as well as inflammation is causally involved in the pathogeneses of amyloidosis [1]. Furthermore, it induces, promotes, or influences susceptibility to several chronic diseases such as atherosclerosis and its clinical complications [3]–[9], type 2 diabetes [10], [11], and various malignancies [12]. The SAA gene family is located within 150 kb at chromosome 11 and comprises of four genes: *SAA1* and *SAA2*, the *bona fide* acute-phase SAA isoforms, *SAA3*, a pseudogene in humans, and *SAA4*, a low level expressed gene coding for C-SAA [13], [14]. A-SAA expression is regulated by a variety of stimuli, including the pro-inflammatory cytokines TNF-α and IL-6, as well as glucocorticoids [15], [16]. Like other acute-phase proteins, A-SAA is expressed primarily by the liver [17]. However, extra-hepatic expression has been reported for different cell lines like epithelial cells, monocyte and macrophage cells, most endothelial cells, adipocytes, atherosclerotic lesions, and smooth muscle cells [17]. Twin studies suggest a substantial genetic contribution to baseline A-SAA concentrations with heritability estimates of 59% (95% confidence interval, 49–67%) [18]. The identification of genetic variants that are predisposed to elevated levels of A-SAA could provide important clues to the immune response pathways involved in the regulation of A-SAA levels which might also be of relevance for related clinical entities. In the past, association analyses between genetic variants and A-SAA levels were limited and restricted to allelic variants of *SAA* genes and protein concentrations [19]–[21].

We therefore conducted the first genome-wide association study on baseline A-SAA concentrations. In a meta-analysis of four genome-wide scans (KORA S4, LURIC, TwinsUK and Sorbs) we included 4,212 participants of European descent. Additionally, in order to account for known gender-specific differences in the regulation of A-SAA [22], [23] we stratified the analysis by gender.

## Results

**Figure 1:**
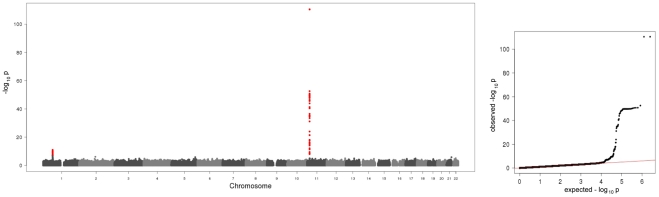
Manhattan plot and quantile-quantile plot of the results of the meta-analysis on baseline A-SAA levels. The Manhattan plot on the left hand side displays all analyzed SNPs with their calculated p-values (p-values below the threshold of genome-wide significance are coloured red). The quantile-quantile plot on the right hand side points out the observed significant associations beyond those expected by chance.

In the present meta-analysis of four genome wide scans 106 SNPs distributed across two regions showed genome-wide significant associations with p-values below the threshold of 5×10^−8^ (Figure 1, Table S1). Table 1 shows study specific results for the top hits within the two regions and three identified subregions (see below) of the meta-analysis as well as an additional region for men in the gender stratified analysis. Genotypic mean levels are provided in Table S2. Results of the single genome-wide studies were consistent across all four studies regarding the direction and magnitude of the effects. In addition, results were consistent between different genotyping technologies (Table S3). No deviations from the Hardy-Weinberg-Equilibrium were observed. The variable of inter-study heterogeneity (I^2^) showed homogeneity at the 1p31 locus. At the 11p15.5-p13 locus we observed I^2^ values that indicated a more distinct heterogeneity. This reflects the relatively large and varying beta values and differences in the minor allele frequency (Table S1). However, taking into account that this locus was clearly significantly associated with A-SAA in all studies included in the meta-analysis, results of the meta-analysis are reported based on a fixed effect model.

**Table 1:** Study-specific results for the hits within the regions/subregions.

SNP	chr	region/subregion	study	effect allele	other allele	imp	effect allele freq	n	beta	se(beta)	p
**rs4150642**	11	11p15.5-p13	KORA S4	G	C	I	0.200	1785	0.529	0.032	1.92E-58
		region	LURIC	G	C	I	0.212	961	0.569	0.075	6.51E-14
			Sorbs	G	C	I	0.212	883	0.524	0.042	1.22E-32
			TwinsUK	G	C	I	0.109	550	0.358	0.050	8.27E-13
			**combined**	**G**	**C**	**-**	**0.193**	**4179**	**0.501**	**0.022**	**3.20E-111**
			validation	G	C	G	0.217	2082	0.473	0.029	7.41E-58
**rs4638289**	11	*SAA1*	KORA S4	A	T	I	0.347	1785	0.323	0.027	4.95E-31
		subregion	LURIC	A	T	I	0.358	961	0.235	0.063	2.17E-04
			Sorbs	A	T	I	0.320	883	0.405	0.044	1.71E-19
			TwinsUK	A	T	I	0.214	514	0.206	0.041	5.70E-07
			**combined**	**A**	**T**	**-**	**0.327**	**4143**	**0.305**	**0.020**	**2.77E-53**
			validation	A	T	G	0.332	2091	0.292	0.025	1.61E-30
**rs4353250**	11	*HPS5/GTF2H1*	KORA S4	T	C	I	0.339	1785	0.278	0.027	1.06E-24
		subregion	LURIC	T	C	I	0.351	961	0.364	0.060	2.21E-09
			Sorbs	T	C	I	0.413	883	0.310	0.035	3.21E-18
			TwinsUK	T	C	I	0.256	577	0.188	0.035	9.01E-08
			**combined**	**T**	**C**	**-**	**0.346**	**4206**	**0.272**	**0.018**	**1.68E-51**
			validation	T	C	G	0.352	2125	0.345	0.025	5.82E-42
**rs2896526**	11	*LDHA/LDHC*	KORA S4	G	A	I	0.177	1785	0.265	0.033	3.99E-15
		subregion	LURIC	G	A	I	0.181	961	0.271	0.079	6.58E-04
			Sorbs	G	A	I	0.163	883	0.226	0.048	3.00E-06
			TwinsUK	G	A	I	0.156	582	0.123	0.042	3.27E-03
			**combined**	**G**	**A**	**-**	**0.172**	**4211**	**0.221**	**0.023**	**4.12E-22**
			validation	G	A	G	0.187	2097	0.216	0.031	8.18E-12
**rs12753193**	1	1p31 (*LEPR)*	KORA S4	A	G	I	0.613	1785	0.103	0.027	1.94E-04
		region	LURIC	A	G	I	0.601	961	0.094	0.064	1.44E-01
			Sorbs	A	G	I	0.599	883	0.177	0.038	4.15E-06
			TwinsUK	A	G	G	0.628	583	0.133	0.033	5.86E-05
			**combined**	**A**	**G**	**-**	**0.609**	**4212**	**0.125**	**0.018**	**1.22E-11**
			validation	A	G	G	0.606	2127	0.085	0.025	8.60E-04
**rs549485**	11	11p14 *(SERGEF)*	KORA S4	T	C	G	0.335	871	0.155	0.040	9.77E-05
males only		region	LURIC	T	C	G	0.368	691	0.105	0.076	1.17E-01
			Sorbs	T	C	G	0.309	361	0.272	0.144	3.18E-05
			**combined**	**T**	**C**	**-**	**0.342**	**1923**	**0.173**	**0.031**	**2.76E-08**
			vlidation*	C	G	G	0.349	1049	0.091	0.035	8.50E-03

*In the validation analysis rs549485 was replaced by rs493767 (r^2^ = 0.961, 3rd lowest p-value within this region in the gender stratified meta-analysis) for technical reasons.

**Figure 2:**
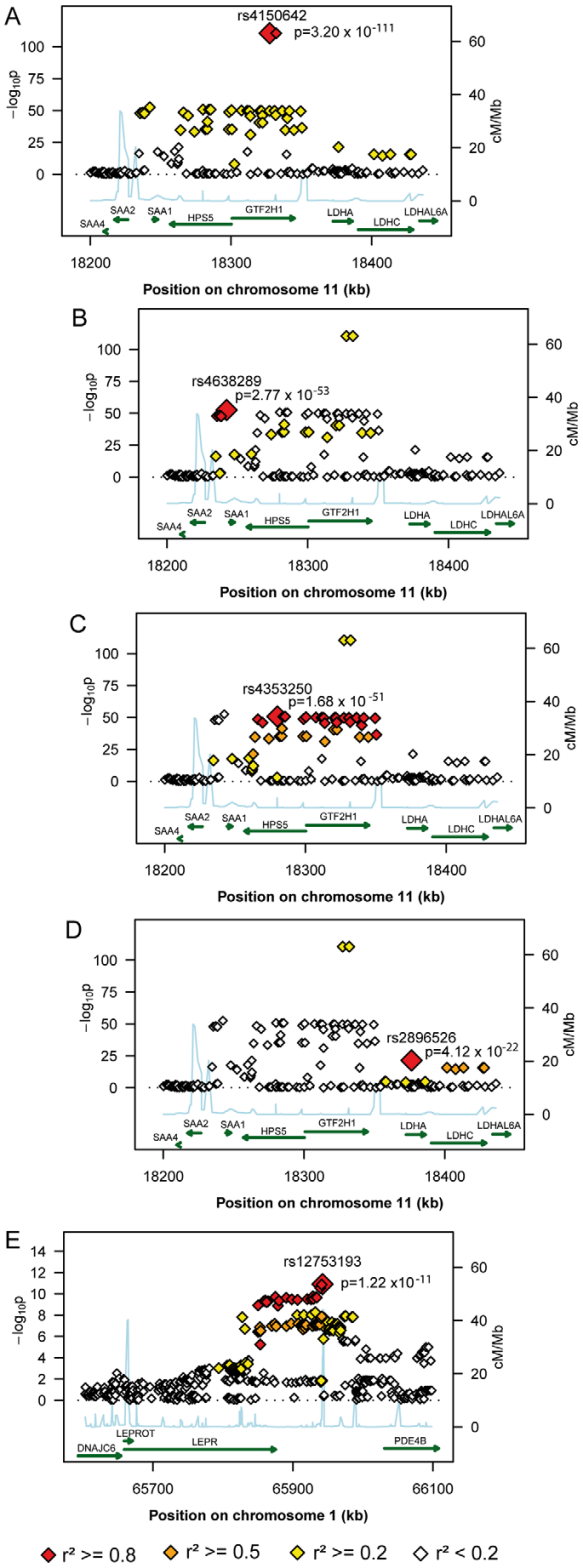
Regional plots of the genetic susceptibility regions/subregions. The regional plots present gene regions and block structures of the region at 11p15.5-p13 (A), the *SAA1* subregion (B), the *HPS5/GTF2H1* subregion (C), the *LDHA/LDHC* subregion (D), and the region at 1p31 (E) and picture the probability values of the significantly associated SNPs, the colour representing the degree of correlation with the top hit of the respective region/subregion.

The first region (193.3 kb of length) resides at 11p15.5-p13 and includes *SAA1* one of the structure genes of A-SAA. Within this region the strongest association was found for two highly correlated intronic polymorphisms of the general transcription factor 2 H1 *(GTF2H1)* gene, rs4150642 (p = 3.20×10^−111^) and rs7103375 (p = 3.26×10^−111^) (Figure 2A). These two top hits show modest correlation (r^2^≤0.376) with other significantly associated SNPs within this region. When the structure of correlation and explained variances within the region were analysed three mostly independent subregions were identified (Table S4, Figure 2B–2D). The first subregion encloses the 5′ end of *SAA1* (Figure 2B) with strongest association for rs4638289 (p = 2.77×10^−53^). The other two subregions harbour the genes Hermansky-Pudlak Syndrome 5 *(HPS5)* and *GTF2H1* (Figure 2C) and lactate dehydrogenase A and C (*LDHA* and *LDHC*) (Figure 2D) with strongest associations for rs4353250 (p = 1.68×10^−51^) and rs2896526 (p = 4.12×10^−22^), two intronic polymorphisms of *HPS5* and *LDHA*, respectively.

The second region was detected at 1p31 (Figure 2E). All 38 significantly associated variants cluster around the 3′ end of the leptin receptor gene *(LEPR)*. The most significantly associated SNP, rs12753193, (p = 1.22×10^−11^) is located downstream of *LEPR*.

All associations were consistent in the KORA S4 validation analyses (Table 1 for the top hits, Table S5 for all SNPs selected for validation).

The entire regression model (including the top SNPs of the two genomic regions (rs4150642 and rs12753193), age, gender and BMI) explains 19.32% of the total variation of A-SAA in our data. With an explained variance of 10.84% for the top SNP (rs4150642) of the 11p15.5-p13 locus (5.57% for rs4638289 of the *SAA1* subregion, 5.34% for rs4353250 of the *HPS5/GTF2H1* subregion, and 2.37% for rs2896526 of the *LDHA/LDHC* subregion; Table S4) and 0.93% for the top SNP (rs12753193) of the 1p31 locus the identified genomic regions account for a major part of such variance.

When the analysis was stratified by gender, one additional SNP (rs549485) located about 350 kb apart from the *SAA1* subregion at 11p14 in the secretion regulating guanine nucleotide exchange factor (*SERGEF*) gene showed a borderline significant association with A-SAA levels in men (p = 2.76×10^−8^) in the meta-analysis. In the validation analysis the association between two highly correlated SNPs within this region (rs493767 and rs550659, r^2^ = 0.961) and A-SAA levels was also borderline significant (p = 8.50×10^−3^ and p = 1.65×10^−2^, respectively). No significant differences between men and women were found within the regions identified in the overall meta-analysis (data not shown).

## Discussion

Based on a meta-analysis of four genome-wide association studies including 4,212 participants of European descent two novel genetic susceptibility regions were identified to be associated with baseline A-SAA concentrations. With 11.68% explained variance in our data, which makes up 19.76% of the total estimated heritability of 59%, these two regions seem to have a major impact on baseline A-SAA concentrations. The region at 11p15.5-p13 accounts for most of the explained variance. Its *SAA1* subregion contains part of a highly conserved region between the two *bona fide* acute-phase structure genes *SAA1* and *SAA2*, which consist of at least 5 and 2 allelic variants, respectively [1], [24]. These two genes are concurrently induced during the acute-phase [1], and cluster within 18 kb of each other in a head to head arrangement [25]. This study is the first presenting the complex genetic architecture of A-SAA levels at this locus. In the identified region, there has been evidence of regulatory elements like C/EBPalpha and C/EBPbeta (http://genome.ucsc.edu), which are necessary for the full responsiveness to IL-1β and IL-6 either alone or in combination [1]. Our finding underlines the high functional potential for this region.

The adjacent *GTF2H1* is a basal transcription factor involved in nucleotide excision repair of DNA and RNA transcription by RNA polymerase II [26]. *HPS5* encodes a protein, which is probably involved in organelle biogenesis associated with melanosomes and platelet dense granule, its mutations lead to a homonymous clinical entity [27]. And *LDHA* and *LDHC*, which are expressed in muscle tissue and in testes, respectively, encode for lactate dehydrogenase, an enzyme that catalyzes the interconversion of lactate and pyruvate [28].

Variants of the *GTF2H1* gene have been recently found to be associated with lung cancer in a Chinese population [29]. Furthermore, it was demonstrated that *LDHA* is involved in tumour genecity and its reduction causes bioenergetic and oxidative stress leading to cell death [30]–[32]. Finally, Kosolowski et al. [33] found *LDHC* to be expressed in several types of tumour cell lines. It is thought, that recurrent or persistent chronic inflammation may play a role in carcinogenesis by causing DNA damage, inciting tissue reparative proliferation and/or by creating an environment that is enriched with tumour-promoting cytokines and growth factors [12]. Furthermore, SAA synthesis could be found in human carcinoma metastases and cancer cell lines [17].

As the approach taken in this study is observational in nature it is not possible to draw causal inferences. For that reason, it could be possible that not genes, but small regulatory elements may be responsible for the findings. This is most likely the case as the identified region contains one structure gene and the adjacent region. In any case, the major impact on baseline A-SAA concentrations demonstrates a key role of the 11p15.5-p13 region in the regulation of inflammation. Therefore, the identification of causal variants and their impact on diseases related to elevated baseline A-SAA concentrations represent promising targets for future functional and epidemiological studies.

The second region was found on chromosome 1p31, harbouring the *LEPR* gene locus. Leptin, an important circulating signal for the regulation of body weight, was found to be correlated with SAA concentrations independently of BMI, and both were expressed in adipose tissue [34]. In the KORA F3 study (Text S1) a moderate but significant correlation was found between circulating A-SAA and leptin concentrations in blood in 181 participants with measurements of both proteins (Spearman correlation = 0.25, p = 7×10^−4^). So far it is unclear whether leptin influences SAA expression directly or via the leptin stimulated cytokines, IL-6 and TNF-α [34]. *LEPR* is a single transmembrane receptor of the cytokine receptor family most related to the gp130 signal-transducing component of the IL-6 receptor, the granulocyte colony-stimulating factor (GCSF) receptor, and the Leukaemia Inhibitory Factor (LIF) receptor, all of which are thought to play an essential role in the inflammatory process [35], [36]. Previous studies have provided evidence of an association of the *LEPR* gene locus with CRP and fibrinogen [37]–[40], which were both correlated with A-SAA in the KORA S4 study (Text S1) (CRP: Spearman correlation = 0.58, p = 3.22×10^−155^, and fibrinogen: Spearman correlation = 0.31, p = 3.89×10^−41^; N = 1734). The finding gives confirmative evidence of the importance of the *LEPR* gene locus for inflammatory processes and the close relationship between leptin, A-SAA, CRP and fibrinogen.

Furthermore, in the gender stratified analysis one region containing *SERGEF* was identified to be presumably associated with A-SAA in men. The adjacency of this identified region to the *SAA* gene family suggests that regulatory elements may be responsible for this signal. However, the association with A-SAA levels was only borderline significant in our study and therefore awaits replication.

Two limitations of our study have to be mentioned. Firstly, due to the restrictions in laboratory methods our analyses were confined to the A-SAA isoforms and did not capture the constitutively expressed C-SAA isoform which might also be of interest, especially when analyzing baseline SAA levels. Secondly, the number of studies with genome-wide data and measured A-SAA levels was limited compared to other genome-wide association studies. Nevertheless, the study had enough power to detect two novel genetic susceptibility regions for A-SAA which explain 19.76% of the total estimated heritability already. Furthermore, results were consistent across all four studies and within different genotyping platforms, the regions are biologically highly plausible, and the results may contribute to future research on the regulation of inflammatory response and its role in related clinical entities.

Taken together, the present meta-analysis is the first whole genome approach to identify genetic variants that are associated with baseline A-SAA concentrations. Two novel genetic susceptibility regions were identified to be associated with baseline A-SAA concentrations. The findings demonstrate a major impact of the 11p15.5-p13 gene region on the regulation of inflammation and suggest a close interplay between leptin, A-SAA, and other acute-phase proteins as well as a larger role of the *LEPR* gene locus in inflammatory processes as it has been assumed in the past.

## Materials and Methods

### Participating studies

The present meta-analysis combined data from four genome-wide scans: one survey of the Cooperative Health Research in the Region of Augsburg (KORA S4), the Ludwigshafen Risk and Cardiovascular Health study (LURIC), the UK Adult Twin Register (TwinsUK) and a self-contained population from Eastern Germany (Sorbs) (). Approval was obtained by each of the local Ethic Committees for all studies and written informed consent was given by all study participants. In total, the meta-analysis included 4,212 individuals (1,928 males, 2,284 females) of European ancestry with measured baseline A-SAA concentrations. For validation analyses we used data of 2,136 participants of the KORA S4 sample, which were not included in the meta-analysis (Text S1). Sample sizes and characteristics of the study participants of the four genome-wide scans and the validation sample are displayed in Table S6.

### Measurement of A-SAA concentrations

In all four studies, study participants were fasting and EDTA plasma samples were analyzed by immunonephelometry on a BNAII device from Siemens, Germany, and well-validated automated microparticle capture enzyme immunoassays [10], [41]. The inter-assay coefficients of variation were below 7% in all four studies.

### Genome-wide genotyping and imputation

For genotyping different platforms as the Affymetrix 500K GeneChip array (Sorbs), Affymetrix 6.0 GeneChip array (KORA S4, LURIC, Sorbs), Illumina HumanHap300 BeadChip (317K) (TwinsUK) and Illumina Human 610K BeadChip (TwinsUK) were used. Quality control before imputation was undertaken in each study separately. Detailed information on genotyping and imputation is reported in Table S7. Imputation based on the HapMap Phase 2 CEU population was performed using IMPUTE [42] in all studies. After imputation all genotype data had to meet the following quality criteria: a minor allele frequency ≥0.01, a call rate per SNP ≥0.9, and r^2^.hat metrics ≥0.40 for imputed SNPs. In total, 2,593,456 genotyped or imputed autosomal SNPs were analyzed in the meta-analysis.

For validation and comparison of genotyping platforms, we selected 27 of the most significantly associated SNPs. Genotyping of these SNPs was performed with the MassARRAY system using the iPLEX technology (Sequenom, San Diego, CA) in the KORA S4 study. The allele-dependent primer extension products were loaded onto one 384-element chip using a nanoliter pipetting system (SpectroCHIP, Spectro-POINT Spotter; Sequenom), and the samples were analyzed by matrix-assisted laser desorption-ionization time-of-flight mass spectrometry (Bruker Daltonik, Leipzig, Germany). The resulting mass spectra were analyzed for peak identification via the SpectroTYPER RT 3.4 software (Sequenom). To control for reproducibility, 9.8% of samples was genotyped in duplicate with a discordance rate of less than 0.5%.

### Genome-wide association analyses and meta-analysis

In each study, linear regression models for all available SNPs have been calculated on ln-transformed A-SAA levels in mg/l. The genetic effect has been assumed to be additive. Adjustment has been made for age, gender, BMI, and study specific covariates, i.e. the Friesinger Score in the LURIC population [43] and a genotyping batch variable in the TwinsUK population. Additionally, this analysis was undertaken stratified by gender. The genome-wide scans were calculated with the analysis software SNPTEST (http://www.stats.ox.ac.uk/~marchini/software/gwas/snptest.html) (KORA S4, LURIC) QUICKTEST (http://toby.freeshell.org/software/quicktest.shtml) (Sorbs) and Merlin (http://www.sph.umich.edu/csg/abecasis/Merlin/) (TwinsUK).

The results of all four genome-wide scans were meta-analysed using a fixed-effects model applying inverse variance weighting with the METAL software (www.sph.umich.edu/csg/abecasis/metal). Study specific results were corrected for population stratification using the genomic control method. For the overall meta-analysis, the inflation factor was 1.009. No further correction was applied.

P-values below the threshold of p = 5×10^−8^, which corresponds to a Bonferroni correction for the estimated number of one million tests for independent common variants in the human genome of European individuals [44], were considered to be significant.

As a measure for between study heterogeneity I^2^ was calculated [45]. Deviations from Hardy-Weinberg-Equilibrium were tested for all identified SNPs by means of the exact Hardy Weinberg test. For the calculation of explained variances, we subtracted the multiple R^2^ value of the covariate model from those of the full model including covariates and top hits of the loci in every single study and assessed the weighted mean (KORA S4, LURIC, and the Sorbs). We tested adjacent regions for independency by analyzing the significance of their top SNPs in a joint model.

### Accession numbers

The OMIM (http://www.ncbi.nlm.nih.gov/omim) accession numbers for genes mentioned in this article are 104750 for *SAA1*, 607521 for *HPS5*, 189972 for *GTF2H1*, 150000 for *LDHA*, 150150 for *LDHC*, 601007 for *LEPR*, and 606051 for *SERGEF*.

## Supporting Information

Table S1 List of all significantly associated SNPs of the meta-analysis.
(0.05 MB PDF)

Table S2 Genotypic mean levels.
(0.02 MB PDF)

Table S3 Comparison between different genotyping technologies in the KORA study.
(0.09 MB PDF)

Table S4 Analysis of the structure of the chromosome 11 region.
(0.04 MB PDF)

Table S5 Results of the validation analyses.
(0.04 MB PDF)

Table S6 Study characteristics of the four studies of the meta-analysis and the validation sample.
(0.04 MB PDF)

Table S7 Study specific information on genotyping and imputation.
(0.06 MB PDF)

Text S1 Study descriptions.
(0.08 MB PDF)
