# Supplementary material for: Genome-Wide Association Study Identifies Two Novel Regions at 11p15.5-p13 and 1p31 with Major Impact on Acute-Phase Serum Amyloid A
Source: PLoS Genet. 2010 Nov 18;6(11):e1001213. doi: 10.1371/journal.pgen.1001213 (PMC2987930; doi:10.1371/journal.pgen.1001213)
Supplement: Table S1 — List of all significantly associated SNPs of the meta-analysis. (0.05 MB PDF) [file pgen.1001213.s001.pdf]

Table S1. List of all significantly associated SNPs of the meta-analysis

SNP	chr	pos	effect allele	other allele	beta	se(beta)	p	n	I²
rs4150642	11	18327474	G	C	0.5012	0.0224	3.20E-111	4179	70.25
rs7103375	11	18332109	G	A	0.5012	0.0224	3.26E-111	4179	70.25
rs4638289	11	18242350	A	T	0.3045	0.0198	2.77E-53	4143	76.45
rs4353250	11	18280093	T	C	0.2719	0.0180	1.68E-51	4206	67.88
rs2061164	11	18285582	T	C	0.2722	0.0180	1.92E-51	4206	67.53
rs10741739	11	18284260	G	T	0.2719	0.0180	2.27E-51	4210	68.05
rs3825025	11	18300398	C	G	0.2711	0.0180	5.32E-51	4210	68.49
rs4150550	11	18307474	C	G	0.2704	0.0180	9.77E-51	4210	68.34
rs4150561	11	18311453	T	C	0.2697	0.0180	1.54E-50	4210	68.18
rs4150562	11	18311571	A	G	0.2697	0.0180	1.58E-50	4210	68.16
rs4150564	11	18311884	G	A	0.2696	0.0180	1.66E-50	4210	68.13
rs4150616	11	18321690	A	T	0.2695	0.0180	1.79E-50	4210	68.02
rs4150641	11	18327315	C	A	0.2695	0.0180	1.79E-50	4210	68.02
rs4150650	11	18330855	G	A	0.2695	0.0180	1.79E-50	4210	68.02
rs4150651	11	18331001	T	C	0.2695	0.0180	1.79E-50	4210	68.02
rs4150661	11	18336205	C	A	0.2694	0.0180	1.80E-50	4210	68.01
rs3740711	11	18336415	T	C	0.2694	0.0180	1.81E-50	4210	68.01
rs4150612	11	18320934	G	T	0.2695	0.0180	1.82E-50	4210	68.02
rs4150610	11	18320716	A	G	0.2694	0.0180	1.83E-50	4210	68.01
rs4150575	11	18313042	C	T	0.2693	0.0180	2.06E-50	4210	67.99
rs9783347	11	18341514	A	G	0.2692	0.0180	2.08E-50	4210	67.98
rs9988866	11	18349559	T	A	0.2673	0.0180	3.68E-50	4210	68.38
rs10741741	11	18298657	T	C	0.2648	0.0179	1.96E-49	4197	63.24
rs1520884	11	18266276	A	G	0.2687	0.0182	3.03E-49	4166	67.39
rs1993373	11	18237211	A	G	0.2860	0.0194	4.21E-49	4206	84.34
rs4150615	11	18321604	C	T	0.2642	0.0180	8.49E-49	4208	65.86
rs7950019	11	18235488	G	A	0.2857	0.0195	1.40E-48	4211	85.10
rs7112278	11	18238492	C	T	0.2828	0.0193	2.09E-48	4157	81.62
rs4150655	11	18331740	A	T	0.2669	0.0185	3.99E-47	4083	67.37
rs4150622	11	18322286	A	G	0.2626	0.0183	8.67E-47	4187	69.91
rs4757638	11	18269529	G	A	0.2619	0.0183	1.12E-46	4127	62.08
rs4150579	11	18313756	A	G	0.2598	0.0182	2.63E-46	4185	67.39
rs4150673	11	18340025	T	G	0.2528	0.0180	1.26E-44	4191	66.21
rs11024614	11	18283334	C	T	0.2338	0.0172	5.05E-42	4205	59.09
rs4150628	11	18322444	G	A	0.2314	0.0172	3.44E-41	4210	58.74
rs4150606	11	18320145	C	A	0.2314	0.0172	3.54E-41	4210	58.73
rs4757645	11	18350421	C	G	0.2284	0.0179	3.26E-37	4092	64.16
rs11024613	11	18283118	T	C	0.2159	0.0172	3.74E-36	4178	55.69
rs3802967	11	18300640	T	C	0.2173	0.0173	5.18E-36	4181	61.73
rs2403254	11	18281722	C	T	0.2150	0.0172	6.07E-36	4168	54.32
rs10832920	11	18298445	A	G	0.2154	0.0172	6.35E-36	4183	55.67
rs4757637	11	18264151	C	A	0.2231	0.0180	2.10E-35	4177	72.63
rs4596	11	18344704	G	C	0.2138	0.0172	2.17E-35	4182	58.32
rs2037867	11	18338597	A	G	0.2137	0.0172	2.17E-35	4182	57.92
rs2305564	11	18274034	T	A	0.2114	0.0173	3.76E-34	4152	60.63
rs4150581	11	18313846	A	G	0.2027	0.0173	7.68E-32	4160	50.29
rs10832918	11	18292260	G	A	0.2437	0.0238	1.09E-24	2722*	0.00
rs3781945	11	18262848	G	A	0.1879	0.0194	3.89E-22	3874	78.09
rs2896526	11	18376382	G	A	0.2211	0.0229	4.12E-22	4211	60.51
rs2271997	11	18259980	C	T	0.1870	0.0211	8.67E-19	3213**	84.34
rs4150563	11	18311678	T	G	0.2284	0.0261	2.15E-18	4024	84.42
rs12218	11	18247897	T	C	0.1650	0.0189	2.18E-18	4104	76.82

rs2045272	11	18234674	T	G	0.1582	0.0188	4.46E-17	4210	55.78
rs2056781	11	18401565	A	G	0.2191	0.0266	1.98E-16	4210	77.72
rs16935424	11	18413313	T	A	0.2191	0.0267	2.13E-16	4210	77.83
rs16935432	11	18427063	A	G	0.2192	0.0267	2.28E-16	4210	77.93
rs12289603	11	18427946	C	T	0.2193	0.0267	2.29E-16	4210	77.98
rs4150667	11	18339487	C	T	0.2011	0.0245	2.31E-16	4015	82.93
rs3740713	11	18407672	C	A	0.2118	0.0268	2.84E-15	4176	77.08
rs7131332	11	18252099	A	G	0.1493	0.0189	3.22E-15	4115	39.79
rs11024600	11	18252386	T	C	0.1468	0.0189	8.22E-15	4210	49.84
rs11024603	11	18262975	G	A	0.1543	0.0215	8.05E-13	4143	43.30
rs35593189	11	18424954	A	G	0.2379	0.0344	4.66E-12	2725*	33.34
rs12291480	11	18262333	G	C	0.1446	0.0231	3.57E-10	4107	34.21
rs1046615	11	18257530	G	A	0.1343	0.0226	2.95E-09	3244**	17.62
rs12416821	11	18257705	A	G	0.1341	0.0226	3.07E-09	3244**	18.03
rs12419588	11	18257706	G	A	0.1341	0.0226	3.07E-09	3244**	18.03
rs2049129	11	18261909	T	C	0.1340	0.0235	1.12E-08	4026	21.75
rs12753193	1	65942267	A	G	0.1248	0.0184	1.22E-11	4212	0.00
rs7541434	1	65941087	C	A	0.1243	0.0184	1.35E-11	4202	0.00
rs7524581	1	65939087	C	T	0.1217	0.0183	2.82E-11	4206	0.00
rs2211651	1	65928609	G	T	0.1132	0.0177	1.66E-10	4210	0.00
rs1805096	1	65874845	G	A	0.1127	0.0177	1.92E-10	4207	0.00
rs6588158	1	65933209	C	T	0.1135	0.0178	1.99E-10	4211	0.00
rs4420065	1	65934049	C	T	0.1133	0.0179	2.23E-10	4212	0.00
rs4655584	1	65928103	T	G	0.1123	0.0177	2.29E-10	4211	0.00
rs4655585	1	65928215	T	C	0.1123	0.0177	2.30E-10	4211	0.00
rs1938492	1	65890417	A	C	0.1122	0.0177	2.33E-10	4210	0.00
rs2889195	1	65929318	C	T	0.1123	0.0178	3.00E-10	4181	0.00
rs11208711	1	65920434	G	T	0.1114	0.0177	3.12E-10	4210	0.00
rs4655582	1	65925951	C	G	0.1114	0.0177	3.20E-10	4211	0.00
rs10789192	1	65898358	G	A	0.1113	0.0177	3.27E-10	4211	0.00
rs12042779	1	65906538	T	C	0.1110	0.0177	3.65E-10	4210	0.00
rs12042807	1	65906640	T	C	0.1110	0.0177	3.65E-10	4210	0.00
rs7516341	1	65860731	T	C	0.1125	0.0180	4.27E-10	4181	18.00
rs7531867	1	65880134	G	A	0.1107	0.0178	4.44E-10	4187	0.00
rs10889569	1	65858782	A	T	0.1122	0.0180	4.71E-10	4183	20.80
rs6588153	1	65864605	T	A	0.1181	0.0190	4.87E-10	4089	0.00
rs6700896	1	65862370	C	T	0.1116	0.0180	5.93E-10	4177	4.96
rs6678033	1	65850212	G	A	0.1097	0.0180	1.21E-09	4178	27.85
rs1892534	1	65878532	C	T	0.1074	0.0177	1.33E-09	4206	0.00
rs4655794	1	65931889	T	C	0.1018	0.0174	5.29E-09	4210	0.00
rs12022410	1	65926521	A	G	0.1001	0.0174	9.51E-09	4211	0.00
rs4655783	1	65915632	T	C	0.1000	0.0174	9.57E-09	4208	0.00
rs11208700	1	65906031	A	T	0.0999	0.0174	9.84E-09	4208	0.00
rs10789198	1	65975343	G	A	0.0991	0.0174	1.20E-08	4183	0.00
rs10889576	1	65941154	C	T	0.1256	0.0221	1.31E-08	4197	0.00
rs4288572	1	65973610	G	A	0.0988	0.0174	1.33E-08	4184	0.00
rs11208728	1	65985631	A	G	0.0985	0.0174	1.48E-08	4182	0.00
rs4655800	1	65984279	A	G	0.0991	0.0175	1.54E-08	4167	0.00
rs6696954	1	65827583	T	G	0.1057	0.0187	1.56E-08	4192	0.00
rs17407727	1	65944594	A	C	0.1038	0.0184	1.80E-08	4197	50.67
rs4425959	1	65940407	A	G	0.1242	0.0221	2.02E-08	4194	0.00
rs17416194	1	65943780	A	G	0.1035	0.0186	2.50E-08	4164	48.62
rs4278348	1	65947983	T	C	0.0947	0.0172	3.54E-08	4209	34.48
rs7532805	1	65955718	G	A	0.0939	0.0172	4.64E-08	4208	35.69

* only KORA S4 and LURIC ** only KORA S4, LURIC, and TwinsUK
