# Supplementary material for: Genome-Wide Association Study Identifies Two Novel Regions at 11p15.5-p13 and 1p31 with Major Impact on Acute-Phase Serum Amyloid A
Source: PLoS Genet. 2010 Nov 18;6(11):e1001213. doi: 10.1371/journal.pgen.1001213 (PMC2987930; doi:10.1371/journal.pgen.1001213)
Supplement: Table S2 — Genotypic mean levels. (0.02 MB PDF) [file pgen.1001213.s002.pdf]

Table S2. Genotypic mean levels

		geometric means (sd) of A-SAA levels				
		**KORA**	**LURIC**	**Sorbs**	**TwinsUK**	**validation sample**
**rs4150642** 11p15.5-p13 locus	GG	7.296 (2.367)	13.052 (3.939)	5.815 (2.114)	7.657 (0.256)	5.933 (2.395)
	GC	4.450 (2.207)	9.573 (3.891)	4.279 (2.036)	7.787 (2.790)	4.192 (2.294)
	CC	2.769 (2.080)	5.151 (3.455)	2.488 (2.061)	5.111(0.471)	2.518 (2.255)
**rs4638289** *SAA1* subregion	AA	4.730 (2.383)	7.594 (3.676)	4.398 (2.182)	7.306 (0.618)	4.469 (2.333)
	AT	3.739 (2.188)	7.599 (3.843)	3.507 (2.073)	6.196 (0.599)	3.371 (2.349)
	TT	2.688 (2.107)	5.430 (3.623)	2.393 (2.112)	5.018 (2.240)	2.511 (2.303)
**rs4353250** *HPS5/ GTF2H1* subregion	TT	5.091 (2.492)	9.969 (3.837)	4.532 (2.361)	7.511 (2.740)	4.805 (2.674)
	TC	3.534 (2.190)	7.843 (3.762)	3.259 (2.102)	5.989 (2.447)	3.456 (2.254)
	CC	2.845 (2.103)	4.914 (3.517)	2.385 (1.981)	5.178 (0.477)	2.463 (2.264)
**rs2896526** *LDHA/LDHC subregion*	GG	4.788 (2.051)	12.469 (4.545)	4.455 (2.383)	6.660 (2.581)	4.227 (2.566)
	GA	4.005 (2.400)	7.793 (3.835)	3.642 (2.142)	6.123 (2.474)	3.607 (2.441)
	AA	3.050 (2.126)	5.974 (3.656)	2.867 (2.146)	5.393 (0.488)	2.861 (2.327)
**rs12753193** 1p31 locus (*LEPR*)	AA	3.673 (2.209)	7.120 (3.710)	3.484 (2.269)	6.221 (2.494)	3.410 (2.468)
	AG	3.241 (2.234)	6.570 (3.758)	2.981 (2.112)	5.398 (0.508)	2.983 (2.342)
	GG	2.961 (2.223)	5.725 (3.888)	2.614 (2.023)	4.742 (0.364)	2.789 (2.240)
**rs549485** 11p14 locus *(SERGEF)* (males only)	TT	3.779 (2.595)	8.583 (4.342)	3.320 (2.925)	NA	3.148 (2.419)*
	TC	3.132 (2.202)	6.119 (3.810)	3.072 (2.103)	NA	2.723 (2.217)*
	CC	2.728 (2.202)	6.093 (4.117)	2.239 (2.200)	NA	2.611 (2.455)*

***** For technical reasons rs549485 was replaced by rs493767 (r^2=0.961, 3rd lowest p-value within this region in the gender stratified meta-analysis) in the validation analysis.
