# Supplementary material for: Genome-Wide Association Study Identifies Two Novel Regions at 11p15.5-p13 and 1p31 with Major Impact on Acute-Phase Serum Amyloid A
Source: PLoS Genet. 2010 Nov 18;6(11):e1001213. doi: 10.1371/journal.pgen.1001213 (PMC2987930; doi:10.1371/journal.pgen.1001213)
Supplement: Table S3 — Comparison between different genotyping technologies in the KORA study. (0.09 MB PDF) [file pgen.1001213.s003.pdf]

Table S3. Comparison between different genotyping technologies in the KORA study

region	SNP	genotyping technology	effect allele	other allele	imp	n	beta	se(beta)	p
11p15.5-p13 locus	rs4150642	Affymetrix 6.0	G	C	I	1785	0.529	0.032	1.92E-58
		Sequenom	G	C	G	1494	0.470	0.032	1.03E-44
	rs7103375	Affymetrix 6.0	G	A	I	1785	0.529	0.032	1.93E-58
		Sequenom	G	A	G	1538	0.473	0.032	1.27E-46
		Illumina 550k	G	A	G	707	0.522	0.050	3.61E-24
SAA1 subregion	rs4638289	Affymetrix 6.0	A	T	I	1785	0.323	0.027	4.95E-31
		Sequenom	A	T	G	1516	0.344	0.028	1.46E-32
	rs1993373	Affymetrix 6.0	A	G	I	1785	0.313	0.027	2.07E-29
		Sequenom	A	G	G	1397	0.348	0.029	1.06E-30
	rs2045272	Affymetrix 6.0	T	G	I	1785	0.199	0.028	2.33E-12
		Sequenom	T	G	G	1526	0.202	0.028	2.12E-12
		Illumina 550k	T	G	G	712	0.115	0.042	5.79E-03
HPS5/ GTF2H1 subregion	rs4353250	Affymetrix 6.0	T	C	I	1785	0.278	0.027	1.06E-24
		Sequenom	T	C	G	1534	0.274	0.029	3.23E-21
	rs9988866	Affymetrix 6.0	T	A	G	1785	0.270	0.027	9.24E-24
		Sequenom	T	A	G	1534	0.275	0.028	1.26E-21
	rs1520884	Affymetrix 6.0	A	G	G	1780	0.271	0.027	1.00E-23
		Sequenom	T	C	G	1459	0.250	0.028	3.24E-19
	rs4150655	Affymetrix 6.0	A	T	G	1659	0.272	0.028	4.51E-21
		Sequenom	A	T	G	1530	0.266	0.029	5.18E-20
	rs4757637	Affymetrix 6.0	C	A	G	1751	0.213	0.026	4.56E-16
		Sequenom	C	A	G	1526	0.232	0.028	6.93E-17
		Illumina 550k	C	A	G	705	0.176	0.041	2.19E-05
	rs4150581	Affymetrix 6.0	A	G	G	1768	0.203	0.026	8.18E-15
		Sequenom	A	G	G	1499	0.210	0.028	3.93E-14
		Illumina 550k	A	G	G	714	0.191	0.040	1.79E-06
	rs11024603	Affymetrix 6.0	G	A	G	1782	0.188	0.032	5.76E-09
		Sequenom	G	A	G	1540	0.181	0.035	1.90E-07
		Illumina 550k	G	A	G	715	0.204	0.051	6.26E-05
LDHA/LDHC subregion	rs2896526	Affymetrix 6.0	G	A	I	1785	0.265	0.033	3.99E-15
		Sequenom	G	A	G	1511	0.252	0.035	1.46E-12
		Illumina 550k	G	A	G	716	0.304	0.053	1.15E-08
	rs12289603	Affymetrix 6.0	A	G	I	1785	0.269	0.039	5.81E-12
		Sequenom	T	C	G	1390	0.212	0.038	1.95E-08
	rs12289603	Affymetrix 6.0	C	T	I	1785	0.269	0.039	6.15E-12
		Sequenom	C	T	G	1535	0.260	0.040	8.56E-11
	rs3740713	Affymetrix 6.0	C	A	G	1749	0.257	0.039	8.35E-11
		Sequenom	G	T	G	1532	0.237	0.041	6.32E-09
		Illumina 550k	G	T	G	702	0.274	0.061	7.54E-06
	rs35593189	Affymetrix 6.0	A	G	G	1764	0.255	0.037	1.07E-11
		Sequenom	A	G	G	1526	0.255	0.040	2.66E-10
1p31 locus (*LEPR*)	rs12753193	Affymetrix 6.0	A	G	I	1785	0.103	0.027	1.94E-04
		Sequenom	A	G	G	1536	0.062	0.029	3.10E-02
		Illumina 550k	A	G	G	715	0.117	0.043	6.02E-03

	rs7524581	Affymetrix 6.0	C	T	I	1785	0.101	0.027	2.45E-04
		Sequenom	C	T	G	1534	0.053	0.029	6.67E-02
		Illumina 550k	C	T	G	715	0.107	0.043	1.20E-02
	rs2211651	Affymetrix 6.0	G	T	I	1785	0.093	0.026	4.17E-04
		Sequenom	G	T	G	1499	0.071	0.029	1.53E-02
		Illumina 550k	G	T	G	715	0.113	0.043	8.17E-03
	rs2889195	Affymetrix 6.0	C	T	G	1754	0.092	0.027	5.90E-04
		Sequenom	C	T	G	1519	0.066	0.029	2.16E-02
		Illumina 550k	C	T	G	716	0.113	0.043	7.76E-03
	rs1892534	Affymetrix 6.0	C	T	G	1779	0.092	0.026	5.27E-04
		Sequenom	G	A	G	1511	0.069	0.028	1.42E-02
		Illumina 550k	G	A	G	716	0.104	0.043	1.45E-02
	rs12022410	Affymetrix 6.0	A	G	I	1785	0.093	0.027	5.66E-04
		Sequenom	A	G	G	1498	0.026	0.029	3.79E-01
		Illumina 550k	A	G	G	715	0.097	0.041	1.84E-02
	rs17407727	Affymetrix 6.0	A	C	G	1773	0.106	0.028	1.45E-04
		Sequenom	A	C	G	1509	0.075	0.030	1.29E-02
		Illumina 550k	A	C	G	716	0.129	0.045	3.75E-03
	rs17416194	Affymetrix 6.0	A	G	G	1744	0.105	0.028	2.04E-04
		Sequenom	A	G	G	1491	0.076	0.030	1.17E-02
		Illumina 550k	A	G	G	716	0.124	0.045	5.60E-03
11p14 locus *(SERGEF)* (males only)	rs493767	Affymetrix 6.0	C	G	G	860	0.140	0.040	5.66E-04
		Sequenom	C	G	G	749	0.142	0.041	5.75E-04
	rs550659	Affymetrix 6.0	G	A	G	873	0.131	0.040	1.16E-03
		Sequenom	G	A	G	742	0.143	0.041	5.97E-04
