# Supplementary material for: Genome-Wide Association Study Identifies Two Novel Regions at 11p15.5-p13 and 1p31 with Major Impact on Acute-Phase Serum Amyloid A
Source: PLoS Genet. 2010 Nov 18;6(11):e1001213. doi: 10.1371/journal.pgen.1001213 (PMC2987930; doi:10.1371/journal.pgen.1001213)
Supplement: Table S4 — Analysis of the structure of the chromosome 11 region. (0.04 MB PDF) [file pgen.1001213.s004.pdf]

Table S4. Analysis of the structure of the chromosome 11 region

		Single SNP model		Multiple SNP model	
**rs4150642** 11p15.5-p13	**beta**	0.501			
	**se(beta)**	0.022			
	**p**	3.20E-111	ΔR² = 0.1084*		
**rs4638289** *SAA1* subregion	**beta**	0.305		0.254*	
	**se(beta)**	0.020		0.024*	
	**p**	2.77E-53	ΔR² = 0.0557*	7.61E-27*	
**rs4353250** *HPS5/GTF2H1* subregion	**beta**	0.272		0.212*	
	**se(beta)**	0.018		0.022*	
	**p**	1.68E-51	ΔR² = 0.0534*	3.64E-22*	
**rs2896526** *LDHA/LDHC* subregion	**beta**	0.221		0.106*	
	**se(beta)**	0.023		0.025*	
	**p**	4.12E-22	ΔR² = 0.0237*	1.81E-05*	ΔR² = 0.0910*

* Values calculated for KORA S4, LURIC, and the Sorbs.
