# Supplementary material for: Genome-Wide Association Study Identifies Two Novel Regions at 11p15.5-p13 and 1p31 with Major Impact on Acute-Phase Serum Amyloid A
Source: PLoS Genet. 2010 Nov 18;6(11):e1001213. doi: 10.1371/journal.pgen.1001213 (PMC2987930; doi:10.1371/journal.pgen.1001213)
Supplement: Table S5 — Results of the validation analyses. (0.04 MB PDF) [file pgen.1001213.s005.pdf]

Table S5. Results of the validation analyses

region	SNP	effect allele	other allele	effect allele freq	n	beta	se(beta)	p
11p15.5-p13 locus	rs4150642	G	C	0.217	2082	0.473	0.029	7.41E-58
	rs7103375	G	A	0.215	2130	0.460	0.028	2.27E-56
SAA1 subregion	rs4638289	A	T	0.332	2091	0.292	0.025	1.61E-30
	rs1993373	A	G	0.327	1939	0.297	0.026	2.04E-29
	rs2045272	T	G	0.581	2118	0.207	0.024	4.09E-17
HPS5/ GTF2H1 subregion	rs4353250	T	C	0.352	2125	0.345	0.025	5.82E-42
	rs9988866	T	A	0.359	2125	0.355	0.025	6.62E-45
	rs1520884	T	C	0.356	2034	0.318	0.024	9.14E-38
	rs4150655	A	T	0.323	2123	0.343	0.025	1.52E-41
	rs4757637	C	A	0.406	2109	0.286	0.025	1.48E-29
	rs4150581	A	G	0.486	2072	0.250	0.024	9.31E-25
	rs11024603	G	A	0.828	2127	0.058	0.031	5.74E-02
LDHA/LDHC subregion	rs2896526	G	A	0.187	2097	0.216	0.031	8.18E-12
	rs2056781	T	C	0.170	1942	0.157	0.032	1.17E-06
	rs12289603	C	T	0.120	2131	0.219	0.034	1.69E-10
	rs3740713	G	T	0.109	2117	0.188	0.036	1.67E-07
	rs35593189	A	G	0.116	2113	0.213	0.035	1.11E-09
1p31 locus (*LEPR*)	rs12753193	A	G	0.606	2127	0.085	0.025	8.60E-04
	rs7524581	C	T	0.624	2123	0.080	0.026	1.85E-03
	rs2211651	G	T	0.591	2079	0.069	0.025	6.89E-03
	rs2889195	C	T	0.603	2116	0.075	0.025	2.94E-03
	rs1892534	G	A	0.627	2090	0.070	0.025	5.39E-03
	rs12022410	A	G	0.410	2089	0.072	0.026	5.32E-03
	rs17407727	A	C	0.288	2085	0.067	0.027	1.55E-02
	rs17416194	A	G	0.278	2063	0.055	0.027	4.20E-02
11p14 locus (*SERGEF)males*	rs493767	C	G	0.349	1049	0.091	0.035	8.50E-03
	rs550659	G	A	0.373	1041	0.084	0.035	1.65E-02
