# Supplementary material for: Genome-Wide Association Study Identifies Two Novel Regions at 11p15.5-p13 and 1p31 with Major Impact on Acute-Phase Serum Amyloid A
Source: PLoS Genet. 2010 Nov 18;6(11):e1001213. doi: 10.1371/journal.pgen.1001213 (PMC2987930; doi:10.1371/journal.pgen.1001213)
Supplement: Table S6 — Study characteristics of the four studies of the meta-analysis and the validation sample. (0.04 MB PDF) [file pgen.1001213.s006.pdf]

Table S6. Study characteristics of the four studies of the meta-analysis and the validation sample

**study**	**KORA S4**	**LURIC**	**Sorbs**	**TwinsUK**	**validation sample**
**N** all (males/females)	1785 (874/911)	961 (693/268)	883 (361/522)	583 (0/583)	2136 (1069/1067)
**Age** mean [years] (sd)	53.91 (8.87)	64.14 (10.80)	47.89 (16.31)	56.74 (10.39)	45.66 (15.76)
**BMI** mean [kg/m²] (sd)	27.72 (4.55)	26.53 (4.52)	27.05 (4.94)	24.67 (4.03)	26.87 (4.75)
**SAA** geometric mean [mg/l]	3.35	6.61	3.09	5.61	3.10
