# Supplementary material for: Genome-Wide Association Study Identifies Two Novel Regions at 11p15.5-p13 and 1p31 with Major Impact on Acute-Phase Serum Amyloid A
Source: PLoS Genet. 2010 Nov 18;6(11):e1001213. doi: 10.1371/journal.pgen.1001213 (PMC2987930; doi:10.1371/journal.pgen.1001213)
Supplement: Table S7 — Study specific information on genotyping and imputation. (0.06 MB PDF) [file pgen.1001213.s007.pdf]

Table S7. Study specific information on genotyping and imputation

study		KORA S4	LURIC	Sorbs	TwinsUK
genotyping platform		Affymetrix 6.0	Affymetrix 6.0	Affymetrix 500K Affymetrix 6.0	Illumina 317k Illumina 610k
calling algorithm		Birdseed	Birdseed	BRLMM Birdseed	Illuminus
QC filters before imputation	individual callrate	≥ 0.93	≥ 0.96	none	≥ 0.95
	SNP callrate	none	none	≥ 0.95	≥ 0.95
	HWE	none	none	> 0.0001	> 0.0001
	MAF	none	none	> 0.01	> 0.01
imputation software		IMPUTE v0.4.2	IMPUTE v0.4.2	IMPUTE v1.0.0	IMPUTE v1.0.0
number of SNPs in meta analysis		2,498,895	2,515,584	2,363,460	2,416,789
inflation factor		0.999	1.025	1.184	1.136
statistical software		SNPTEST v2.1.0	SNPTEST v2.1.0	QUICKTEST v0.95	Merlin v1.1.2
