# Supplementary material for: Genome-Wide Association Study Identifies Two Novel Regions at 11p15.5-p13 and 1p31 with Major Impact on Acute-Phase Serum Amyloid A
Source: PLoS Genet. 2010 Nov 18;6(11):e1001213. doi: 10.1371/journal.pgen.1001213 (PMC2987930; doi:10.1371/journal.pgen.1001213)
Supplement: Text S1 — Study descriptions. (0.08 MB PDF) [file pgen.1001213.s008.pdf]

Text S1. Study descriptions

KORA studies

The Cooperative Health Research in the Region of Augsburg (KORA) study is a series of independent population-based epidemiological surveys and follow-up studies of participants living in the region of Augsburg, Southern Germany. All participants are residents of German nationality identified through the registration and informed consent has been given by all participants [1]. The study has been approved by the local ethics committee. The present study includes data of the KORA S4 survey (1999/2000), the follow-up study F4 (2006-2008) and the follow-up study of KORA S3 survey (1994/1995) F3 (2004/2005). For the genome-wide association study we genotyped 1,814 randomly selected participants of KORA F4 with Affymetrix 6.0. A-SAA measurements were available for 1,785 of these participants. Additionally, 27 SNPs, which were identified in the meta-analysis, were genotyped with the MassARRAY system using the iPLEX technology (Sequenom) in all participants with A-SAA measurements of the KORA S4 study (N=3,677) thus providing one sample for validation (N=2,136) and one sample for the comparison of the genotyping technologies (N=1,541, corresponding to the sample genotyped with Affymetrix 6.0). For the comparison of the genotyping technologies, we used an additional subsample of 717 KORA S4 participants with genotyping data available from the Illumina 550K platform. Correlation analysis was conducted in all participants of S4 (N=4,261) and F3 (N=3,006) where measurements of SAA, CRP, fibrinogen and leptin were available. Methods for measurement have been described in detail elsewhere [2,3].

LURIC study

The **Lu**dwigshafen **Ri**sk and **C**ardiovascular Health (LURIC) study includes consecutive white patients of Caucasian origin (17 to 92 years of age) hospitalized for coronary angiography between June 1997 and May 2001 [4]. The study was approved by the ethics review committee at the “Landesärztekammer Rheinland-Pfalz”. Written informed consent was obtained from each of the participants. Clinical indications for angiography were chest pain or non-invasive tests consistent with myocardial ischemia. To limit clinical heterogeneity, individuals suffering from acute illness other than acute coronary syndromes, chronic non-cardiac diseases and a history of malignancy within the five past years were excluded. The present study includes data of 1002 participants for whom Affymetrix 6.0 genotyping data was available.

Sorbs study

All subjects are part of a sample from an extensively phenotyped self-contained population from Eastern Germany, the Sorbs (PMID: 19584900; PMID: 19729412). The Sorbs are of Slavonic origin, and lived in ethnic isolation among the Germanic majority during the past 1100 years. At present, about 1000 Sorbian individuals are enrolled in the study. Sampling comprised unrelated subjects as well as families. Mean IBD sharing in the pairwise comparison was 0.008, median $<10^{-6}$ (25% percentile $<10^{-6}$, 75% percentile: 0.012). Extensive phenotyping included standardised questionnaires for past medical history and family history, collection of anthropometric data and a 75g-Glucose-tolerance-test. The study was approved by the ethics committee of the University of Leipzig and all subjects gave written informed consent before taking part in the study.

TwinsUK study

The TwinsUK adult twin registry based at St Thomas' Hospital in London is a volunteer cohort of over 10,000 twins recruited among the general population. Twins largely volunteered unaware of any their phenotypic status in relationship to SAA or any other phenotypic trait of interest to the authors and they gave fully informed consent under a protocol reviewed by the St Thomas' Hospital Local Research Ethics Committee. All participants in the TwinsUK study were female and SAA measurements were obtained as described in the main methods section.

References

1. Wichmann HE, Gieger C, Illig T, MONICA/KORA Study Group (2005) KORA-gen - resource for population genetics, controls and a broad spectrum of disease phenotypes. Gesundheitswesen 67: 26-30.

2. Müller S, Martin S, Koenig W, Hanifi-Moghaddam P, Rathmann W, et al. (2002) Impaired glucose tolerance is associated with increased serum concentrations of interleukin 6 and co-regulated acute-phase proteins but not TNF-α or its receptors. Diabetologia 45: 805-812.

3. Karakas M, Zierer A, Herder C, Baumert J, Meisinger C, et al. (2010) Leptin, adiponectin, their ratio and risk of Coronary Heart Disease: results from the MONICA/KORA Augsburg Study 1984-2002. Atherosclerosis 209: 220-225.

4. Winkelmann BR, März W, Boehm BO, Zotz R, Hager J, et al. (2001) Rationale and design of the LURIC study--a resource for functional genomics, pharmacogenomics and long-term prognosis of cardiovascular disease. Pharmacogenomics 2: 1-73.
